# Probing the function of C-terminal region of recombinant α-amylase BmaN1 from *Bacillus megaterium* NL3

**DOI:** 10.1128/spectrum.03351-23

**Published:** 2024-08-30

**Authors:** Fina Khaerunnisa Frima, Muhammad Akbar Thufail, Indri Novia Madhani, Zahrotun Nafisah, Sofi Siti Shofiyah, Ayra Ulpiyana, Fernita Puspasari, Reza Aditama, Ihsanawati Ihsanawati, Dessy Natalia

**Affiliations:** 1 Biochemistry and Biomolecular Engineering Research Division, Faculty of Mathematics and Natural Sciences, Institut Teknologi Bandung, Bandung, Indonesia; 2 Department of Chemistry, Faculty of Science, Institut Teknologi Sumatera, Lampung Selatan, Indonesia; 3 Department of Chemistry, Faculty of Science and Marine, Universitas Oso, Pontianak, Indonesia; 4 Biosciences and Biotechnology Research Center, Institut Teknologi Bandung, Bandung, Indonesia; Center of Innovative and Applied Bioprocessing, Mohali, Punjab, India

**Keywords:** *Bacillus megaterium *NL3, α-amylase BmaN1, GH13_45 subfamily, C-terminal region

## Abstract

The α-amylase BmaN1 from *Bacillus megaterium* NL3 is a member of GH13_45 subfamily that has a conserved C-terminal region of approximately 30 residues. This region features a motif of five aromatic amino acids predicted to play a role in starch binding. This study aimed to unravel the role of the C-terminal region in starch hydrolysis. The full-length and C-terminally truncated forms of BmaN1 (BmaN1∆C) were expressed in *Escherichia coli* ArcticExpress (DE3), resulting in proteins with molecular weights of 56 kDa and 49 kDa, respectively. They exhibited comparable enzymatic activity in the hydrolysis of soluble starch, displaying versatility across a wide range of pH values, temperatures, and NaCl concentrations. BmaN1 and BmaN1∆C activities were inhibited by acarbose and were reduced by SDS and EDTA. In terms of binding and degrading the starch granules, BmaN1∆C showed lower affinity and activity in comparison to BmaN1. Our study indicates that the C-terminal region of BmaN1 significantly enhances its binding affinity and degrading the raw starches.

**IMPORTANCE:**

α-Amylase (EC 3.2.1.1) stands as an endo-acting enzyme, essential for catalyzing the hydrolysis of α-1,4 glycosidic bonds within starch molecules. The relevance of α-amylases in biotechnological applications is substantial, constituting approximately 30% of the global enzyme market. Among these enzymes, BmaN1 was the first α-amylase identified to possess distinct catalytic residues within the GH13 family. BmaN1 from *B. megaterium* NL3 belongs to the GH13_45 subfamily. This subfamily is characterized by a conserved C-terminal region consisting of approximately 30 residues that contains a motif of five aromatic residues predicted to be involved in starch binding. Our study shows that the C-terminal effectively contributes to binding and degrading the raw starch granules. This pioneering research on BmaN1 expands our understanding of α-amylases and holds promise for innovative biotechnological advancements.

## INTRODUCTION

α-Amylase (EC 3.2.1.1) is one of the most prominent enzymes involved in the hydrolysis of internal α-1,4-glycosidic bonds in starch and related polysaccharides (1, 2). According to the sequence-based classification system for carbohydrate-active enzymes (CAZy), α-amylase is the primary representative enzyme within the glycoside hydrolase 13 (GH13) family (3–6). Members of the GH13 family share several distinct properties, signifying their shared characteristics in both structure and function. These criteria include the ability to hydrolyze α-glycosidic bonds using the α-retaining mechanism, possession of a (β/α)_8_-barrel as the structure of the catalytic domain, exhibition of 4–7 conserved sequence regions (CSRs) within the catalytic domain, and sharing the identical catalytic triad (7–10). As of December 2023, α-amylases have diversified into various subfamilies, each distinguished by unique characteristics, such as GH13_1, GH13_5, GH13_6, GH13_7, GH13_15, GH13_24, GH13_27, GH13_28, GH13_36, GH13_37, GH13_39, GH13_41, GH13_42, GH13_43, GH13_45, GH13_46, and GH13_47 (10–12). These subfamilies, as documented on www.cazy.org, highlight the diverse evolutionary adaptations and functions within the GH13 family.

The GH13_45 subfamily is composed of bacterial α-amylases, encompassing approximately 862 amino acid sequences (www.cazy.org; December 2023). Phylogenetic analysis of α-amylases within the GH13 family reveals that GH13_45 is closely related to subfamilies GH13_46, GH13_36, and GH13_37. Members of the GH13_45 subfamily share three additional distinctive sequence-structural features, a pair of adjacent tryptophan residues positioned between CSR-V and CSR-II, the LPDlx motif within CSR-V, and a C-terminal region of approximately 30 residues containing five conserved aromatic residues (13). Within the GH13_45 subfamily, only a limited subset of members has been characterized to date. Those are AmyB from *Anaerobranca* (14); ASKA, ADTA, and AGXA from *Anoxybacillus* (15, 16); Pizzo^T^, GTA, Gt-AmyII, and AmyP from *Geobacillus thermoleovorans* (17–19); BaqA from *Bacillus aquimaris* MKSC 6.2 (20, 21); Amy-E from *Exiguobacterium* (22); and BmaN1 from *B. megaterium* NL3 (23).

BmaN1 features a triad catalytic residue, in which Asp203 is the nucleophile, Glu231 serves as the proton donor, and His294 is predicted as the transition state stabilizer (23). Notably, BmaN1 differs from other members of the α-amylase GH13_45 subfamily by having His294 instead of the conserved aspartate. In addition, BmaN1 together with BaqA and other α-amylases within the GH13_45 subfamily have a unique C-terminal region. The C-terminal truncated GTA and Gt-amy (GH13) have more compact structures and higher thermostability (18, 24). Despite the importance of conserved motifs in the C-terminal domain of GH13_45, the functional role of this domain is still relatively unexplored at present.

The primary objective of this study was to unravel the role of the C-terminal region of BmaN1 concerning enzyme stability and activity in starch hydrolysis. To accomplish this goal, we carried out a comprehensive exploration of the biochemical characteristics of a full length and a C-terminal truncated recombinant enzyme, namely, BmaN1 and BmaN1ΔC, respectively. This truncated enzyme lacked the 45 residues at the C-terminal region. The investigation focused on assessing the enzyme performance in the hydrolysis of both soluble and raw starch. By examining both the full-length and truncated forms, the study aimed to understand the specific contributions of the C-terminal region to the enzyme stability and activities in starch hydrolysis. The results of these studies offer valuable insights into the significance of the C-terminal region of BmaN1.

## MATERIALS AND METHODS

### Materials

The chemicals utilized in this study were of analytical grade and sourced from reputable commercial suppliers, namely, Sigma and Merck (USA). Several enzymes were used in this study, namely, Dream *Taq* polymerase (Thermo Fisher, USA), the restriction endonucleases *NdeI* and *XhoI* (NEB, USA), and T4 DNA ligase (Thermo Fisher, USA). For the purpose of electrophoresis, a 1 kb DNA ladder was purchased from Thermo Fisher (USA) and ExcelBand 3-color Broad Range Protein Marker was obtained from SMBIO (Taiwan). The primers for PCR were synthesized by Macrogen Inc. (Korea). In the protein purification process, Nickel-NTA agarose from Qiagen (The Netherlands) was employed. To determine protein concentration and for electrophoresis analysis, we utilized a HiMedia (India) reagent and a solution kit from Bio-Rad (USA).

### Strains and plasmids

*Escherichia coli* TOP10F′ (Thermo Fisher, USA) served as the host for cloning purposes, while *E. coli* ArcticExpress (DE3) (Stratagene, USA) was employed for gene expression. Plasmids pGEM-T (Promega, USA) and pET30EZ were utilized for gene cloning and gene expression, respectively. The cells were cultured in Luria-Bertani (LB) medium (comprising 1% (wt/vol) tryptone, 1% (wt/vol) NaCl, and 0.5% (wt/vol) yeast extract), supplemented with the appropriate antibiotics for selection of pGEM-T and pET30EZ, respectively. For solid media, 2% (wt/vol) agar was added to the LB medium.

### Methods

#### Construction of pET30EZ-bmaN1

A plasmid concentration of approximately 20 ng/µL for pGEMT-bmaN1 was used as a template to amplify the *bmaN1* gene with the signal peptide sequence (1–22) deleted. Oligonucleotide primers (pEZ-bmaN1-F 5′ GGA GAT ATA CAT ATG CAA GAT CAT AAA GAT ATA CGT GAT GAG G 3′ and pEZ-bmaN1-R 5′ ACG TGG AAC TAA CTC GAC CGA CGC GCT GTC CTT TTT AC 3′) were utilized in *bmaN1* gene amplification with a cycling protocol involving predenaturation at 94°C for 2 minutes. This was followed by 30 cycles of denaturation at 94°C for 30 seconds, annealing at 50–67°C for 30 seconds, extension at 72°C for 90 seconds, and a final extension of 5 minutes at 72°C. The resulting amplicon was subcloned into the linear vector pET30EZ, a modified pET-30a without oligonucleotide encoding 12 amino acid residues at the N-terminus and containing a thrombin site and a 6×His-tag (LVPRGSHHHHHH) at the C-terminal end. The ligation product was introduced into competent *E. coli* TOP10F′ cells and subsequently sequenced at Macrogene (Singapore).

#### C-terminal truncation and construction of pET30EZ-bmaN1ΔC

To generate C-terminal truncation involving the removal of the 45 residues from BmaN1, we employed polymerase chain reaction. Oligonucleotide primers Bman1∆C_H6_F (5′ CATATGCAAGATCATAAAGATATACGTGATGAGG 3′) and Bman1∆C_H6_R (5′ CTCGAGCTTTTTCACTGCTTTATATACATTTGTG 3′) were utilized to amplify the gene of *bmaN1∆C* (BmaN1 with C-terminal truncation) in a Thermal Cycler (BioRad, USA). The cycling conditions involved initial denaturation at 95°C for 3 minutes, followed by 29 cycles of denaturation at 95°C for 30 seconds, annealing at 66.7°C for 30 seconds, extension at 72°C for 30 seconds, and a final extension of 5 minutes at 72°C. The resulting amplicon was cloned into pGEM-T and sequenced. The pGEM-T constructs of *bmaN1∆C* were then digested with *Nde*I and *Xho*I and subcloned into linear pET30EZ vector to generate the pET30EZ-bmaN1∆C recombinant vector. The ligation product was introduced into competent *E. coli* TOP10F′ cells. Positive clones, confirmed through double digestion of the constructs by *Nde*I and *Xho*I, were subsequently sequenced for validation.

### Bioinformatic analysis

The process began with retrieval of the amino acid sequence of α-amylase BmaN1 from *B. megaterium* NL3 via UniProt (Accession Number: T1SIF2). An alignment of the amino acid sequences between BmaN1 and the characterized GH13_45 subfamily revealed a highly conservation spanning 45 amino acids (positions 461–504). This conserved sequence includes five aromatic residues and is specifically located in the C-terminal region of the protein. The transmembrane helix and protein topology analysis of the amino acid sequence of BmaN1 was conducted using CCTOP (https://cctop.ttk.hu/) (25). The 45 residues at C-terminal region of BmaN1 contain a transmembrane helix at positions 467–482 (IAAVAAVYTGFGIFLY).

Subsequently, the amino acid sequence of BmaN1 was converted into the .pdb file format for modeling purposes using AlphaFold2 (https://alphafold.ebi.ac.uk/) (26). To generate the BmaN1∆C structure, we removed the transmembrane region (461–504). The prepared protein structure underwent simulation setup using the PlayMolecule Server (http://playmolecule.org). The structural integrity was then assessed through PROCHECK on the UCLA-SAVES server (https://saves.mbi.ucla.edu/). An input file containing Molecular Dynamics (MD) simulation parameters for the tleap command were meticulously prepared. These parameters included solvation with water using the TIP3P model, maintaining a box size of 12 Å, and introducing Na^+^ and Cl^−^ counter-ions based on varying concentrations (0.0, 0.5, 1.0, 2.0, and 3.0 M). MD simulations were executed using AMBER18 software with the FF14SB force field. A minimization step was performed at 323 K, the optimal temperature for starch hydrolysis by BmaN1, using the steepest descent method for 2,000 steps. The initial equilibration was achieved using isochoric-isothermal (NVT) conditions to model a system where expansion remains constant for 500 steps. The final equilibrium was achieved using isobaric-isothermal (NPT) conditions, representing various system conditions with diverse volume expansion and constant pressure. The production step spanned 100 ns across the entire protein system, employing a time step of 2 fs. A cutoff value of 1.2 nm was applied to alleviate computational load. Energy data and coordinates were recorded every 10 ps during the simulation. The resulting simulation trajectory was subjected to analysis using cpptraj and Visual Molecular Dynamics (VMD). The assessment involved root mean square deviation (RMSD) to evaluate the three-dimensional structure's compactness and root mean square fluctuation (RMSF) to measure residue flexibility in different salt concentrations.

Multiple sequence alignments of BmaN1 (GenBank ID: AGT45938.1) and other α-amylases were conducted using BioEdit (27). Amino acid sequences of α-amylases from various subfamilies of GH13 were collected through the CAZy database (www.cazy.org) and GenBank database (https://www.ncbi.nlm.nih.gov/genbank/) (28). The sequence logo representing the GH13_45 characteristics was generated through the WebLogo 3.0 server (http://weblogo.threeplusone.com/) (29).

### Expression and purification BmaN1 and BmaN1ΔC

Plasmids pET30EZ-bmaN1 and pET30EZ-bmaN1ΔC were individually introduced into *E. coli* ArcticExpress (DE3) through transformation. Recombinant enzyme expression was induced by adding 0.5 mM isopropyl ß-D-1-thiogalactopyranoside (IPTG), followed by further incubation at 10°C and 150 rpm for 24 hours. The cell pellet was resuspended in binding buffer (50 mM HEPES, 500 mM NaCl, 10 mM imidazole, pH 8.0) and subjected to sonication for 15 minutes with a pulse and interval time of 33 seconds each. Both enzymes were subsequently purified using Ni^2+^-NTA resin affinity chromatography. The purification process for the BmaN1 protein involved initial purification under denaturing conditions, followed by refolding through a decreasing urea gradient to obtain the active enzyme. The truncated variant was purified directly under native conditions. Elution of the recombinant α-amylases was achieved using 250 mM imidazole, and the purified enzymes were analyzed on sodium dodecyl sulfate polyacrylamide gel electrophoresis (SDS-PAGE). Active fractions were dialyzed against 100 mM Tris buffer (pH 8) and stored at 4°C. The protein concentration was determined using the Bradford assay (30). The purified enzymes were then used for subsequent biochemical characterizations.

### α-Amylase assay

The α-amylase assay involved the hydrolysis of 2% soluble starch, prepared in a universal MES buffer (25 mM L-malic acid, 50 mM MES, and 50 mM Tris) at pH 6.5. This mixture was incubated at 50°C for 10 minutes. The liberated reducing sugars were then quantified using the 3,5-dinitrosalicylic acid reagent**,** as described by Miller (31). One unit of α-amylase activity is defined as the amount of enzyme capable of producing 1 µmol of reducing sugars (as glucose) per minute under the specified assay conditions.

### Biochemical characterization of the recombinant BmaN1 and BmaN1ΔC

The determination of the optimal pH for the activity of the purified enzymes involved incubating the reaction mixtures at 50°C in universal MES buffer within the pH range of 4.5–8.5. Additionally, the optimal temperature for amylolytic activity was investigated by incubating the reaction mixture (maintained at pH 6.5) at temperatures ranging from 30°C to 80°C. The impact of NaCl concentration on the hydrolysis of 2% soluble starch by both enzymes was assessed at total NaCl concentrations of 0.5, 1.0, 2.0, and 3.0 M, respectively. The stability of the recombinant enzymes was evaluated by preincubating them at 50°C for varying time intervals. Furthermore, the inhibitory effect of acarbose (at concentrations ranging from 10 to 50 µM) on enzyme activity was determined by measuring the residual activity after incubation with acarbose under optimum conditions. The influence of EDTA and SDS on the activity of both enzymes was investigated by introducing these substances into the reaction at different concentrations.

To explore the kinetic parameters of BmaN1 and BmaN1ΔC, we used various concentrations of soluble starch (ranging from 10 to 100 mg/mL) in a universal MES buffer at pH 6.5 under optimal conditions. Kinetic parameters, *V*_max_, *K*_m_, *k*_cat_*,* and *k*_cat_*/K*_m_ were determined by fitting of the initial reaction rates against concentrations of soluble starch using the Michaelis-Menten (MM) equation.

### Raw corn starch adsorption

Both purified BmaN1 and BmaN1ΔC (at a concentration of 0.08 mg/mL) incubated with 5% (wt/vol) corn and cassava starch granules at in 100 mM Tris buffer at pH 8.0. The incubation occurred at 4°C for 2 hours with continuous shaking at 200 rpm and then centrifuged at 12,000 rpm for 10 minutes to precipitate the starch and bound protein. The residual unbound protein was determined using the Bradford assay. The percent bound protein = (original protein ‒ unbound protein)/original protein × 100.

### Determination of raw starch digestibility

To assess the digestion of raw starch, we combined both enzymes (at a concentration of 0.65 µg/µL) with 5% (wt/vol) of various types of raw starch (corn and cassava) in a final volume of 0.1 mL in universal MES buffer at pH 6.5. The resulting mixture was incubated for 24 hours with continuous shaking at 50°C (175 rpm). Following incubation, the mixture underwent centrifugation, and the quantity of reducing sugar released during the enzymatic activity was measured using the DNS method. The degree of hydrolysis (DH) was determined using the following equation:


$$DH\left(\%\right)=\frac{H_{1}}{H_{0}}\times 100$$

where $H_{1}$ was reducing sugar produced by enzymatic hydrolysis and $H_{0}$ was reducing sugar produced by acidic hydrolysis.

### Statistical analysis

The α-amylase assays were carried out parallel in triplicate sets. The values were determined and are presented as the mean ± standard deviation. The all statistical analysis were performed using GraphPad Prism 9 (32).

### Scanning electron microscopy

The pellet from the mixture of raw starch (corn and cassava) and enzymes (0.65 µg/µL) was washed with 95% ethanol then dried at 37°C overnight. The sample was put on the surface of carbon tape and coated with gold by ion sputtering (MC1000; Hitachi High-Technologies Corporation, Japan) and photographed using scanning electron microscopy (SEM) (Hitachi SU3500; Hitachi High-Technologies Corporation, Japan) operated at 10 kV.

## RESULTS

### Expression and purification of BmaN1 and BmaN1ΔC

**Fig 1:**
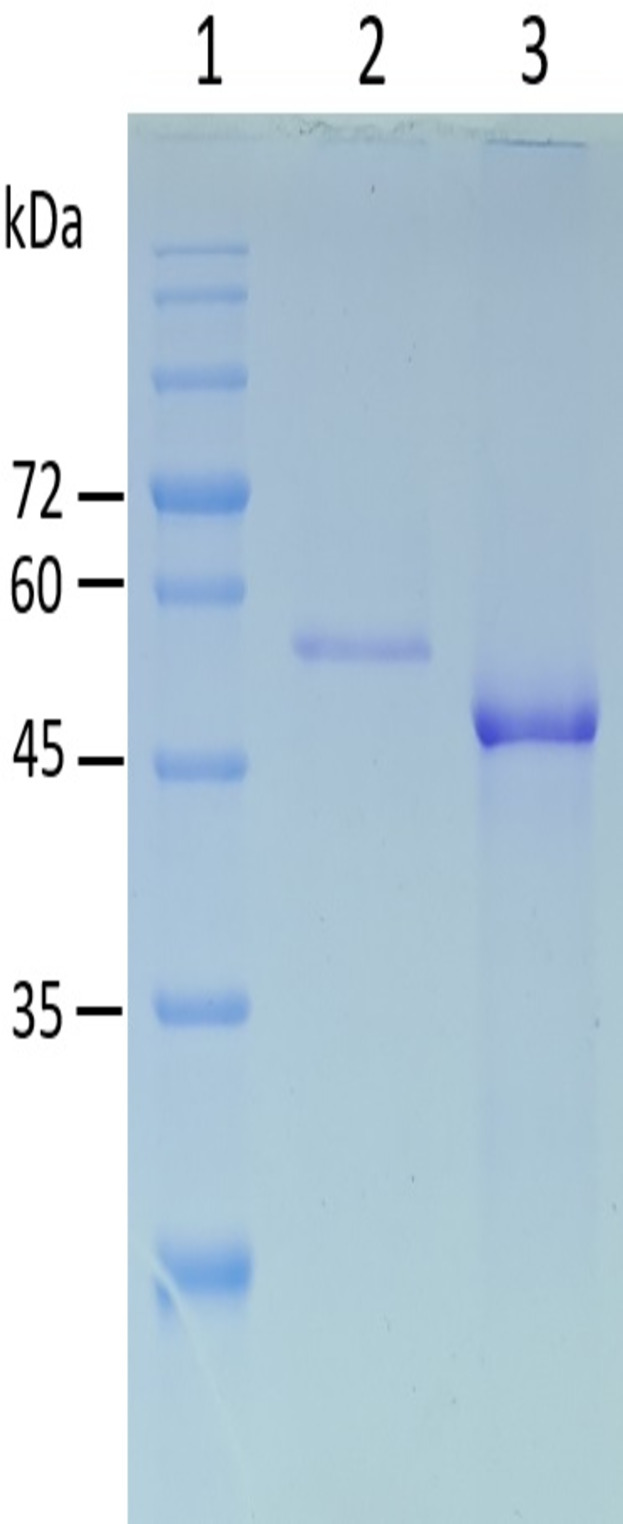
SDS-PAGE analysis of BmaN1 and BmaN1ΔC. Lane 1: protein molecular weight marker (kDa); Lane 2: purified recombinant BmaN1 obtained through the Ni-NTA affinity chromatography; Lane 3: purified recombinant BmaN1ΔC eluted from the Ni-NTA column.

The pET30EZ-bmaN1 and pET30EZ-bmaN1ΔC recombinant plasmids were introduced into *E. coli* ArcticExpress (DE3). BmaN1 was expressed as inclusion bodies with a molecular weight of 56 kDa, as confirmed by SDS-PAGE analysis (Fig. 1). To obtain an active BmaN1, we refolded the inclusion bodies by urea as a denaturing agent in the Ni-NTA affinity chromatography. The refolded BmaN1 had a specific activity of 1.54 ± 0.23 U/mg. The α-amylase BmaN1ΔC was produced as a soluble protein with a molecular weight of 49 kDa (Fig. 1) and purified to homogeneity with a 12-fold purification. BmaN1ΔC displayed amylolytic specific activity of 1.69 ± 0.16 U/mg.

### Biochemical characterization of the recombinant BmaN1 and BmaN1ΔC

**Fig 2:**
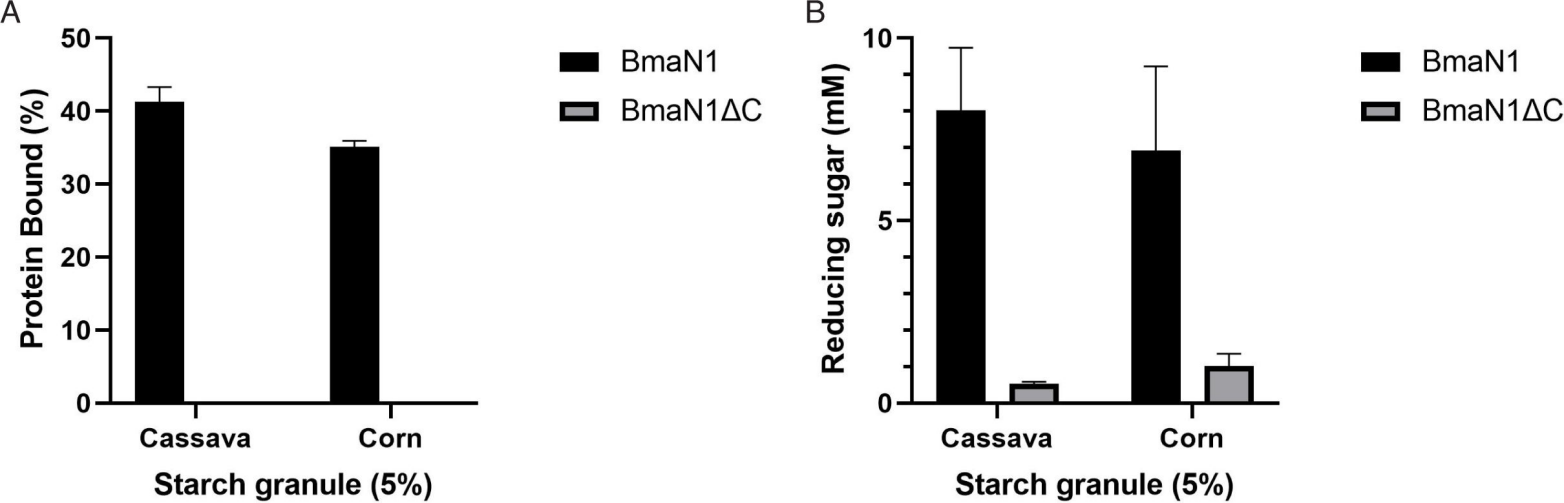
Effect of the C-terminal region of BmaN1 on raw starch adsorption and hydrolysis. (**A**) Protein binding to raw starch; (**B**) hydrolysis of raw starch by BmaN1 and BmaN1ΔC.

**Fig 3:**
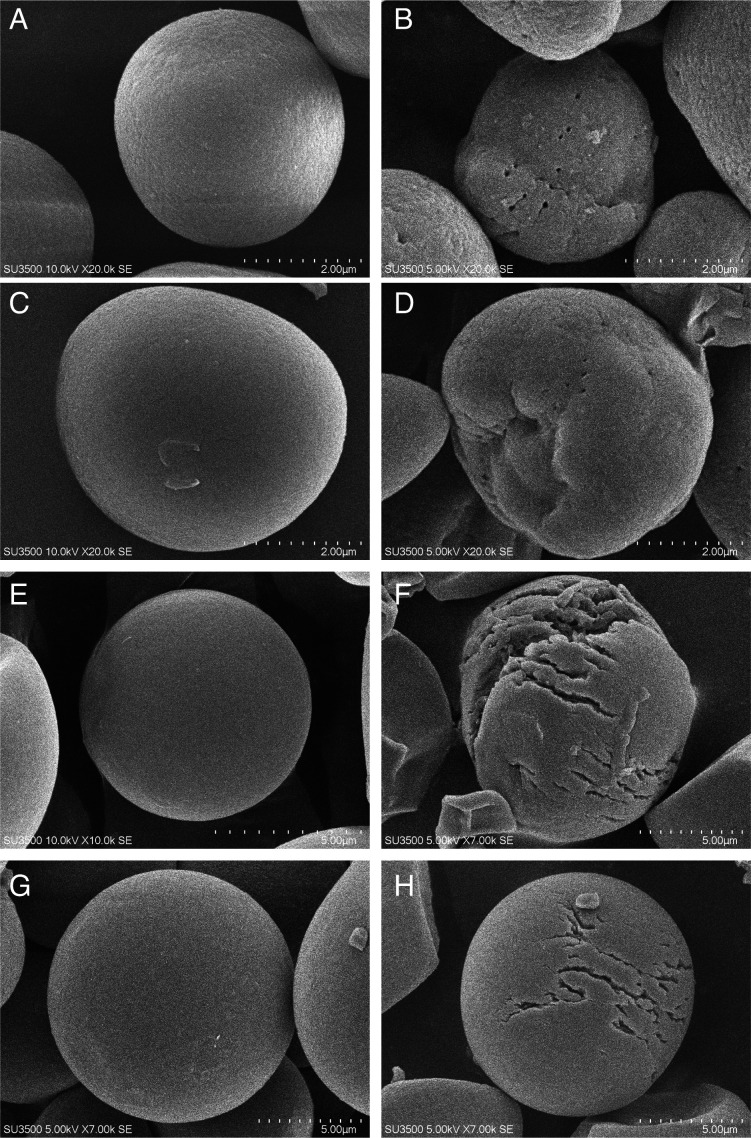
Scanning electron microscopy of untreated and treated raw starch granules by BmaN1 and BmaN1ΔC. (**A**) BmaN1 untreated corn granule, (**B**) BmaN1 treated corn, (**C**) BmaN1ΔC untreated corn, (**D**) BmaN1ΔC treated corn, (**E**) BmaN1 untreated cassava granule, (**F**) BmaN1 treated cassava, (**G**) BmaN1ΔC untreated caasava, (**H**) BmaN1ΔC treated cassava.

BmaN1 demonstrated the ability to bind and degrade corn and cassava granules, resulting in the production of reducing sugars (Fig. 2). The adsorption of BmaN1 to 5% cassava and 5% corn raw starch was observed to be protein bound of 41% and 34%, respectively. The amounts of reducing sugar generated from corn and cassava granules were 6.92 ± 2.30 mM and 8.02 ± 1.71 mM, corresponding to hydrolysis rates (degree of hydrolysis/DH) of 3.1% and 3.6%, respectively. Meanwhile, BmaN1ΔC demonstrated an inability to adsorb to raw starch and displayed much lower activity towards raw starch when compared to BmaN1 (Fig. 2). The enzymatic capability of both BmaN1 and BmaN1ΔC to hydrolyze the raw starch was further confirmed through scanning electron microscope (SEM) analysis (Fig. 3). The untreated corn and cassava starch exhibited a smooth granule surface (Fig. 3A, C, E and G). However, both granules treated with enzymes have distinct structural changes. The corn raw starch had holes on the granule surface (Fig. 3B and D), while the raw cassava surfaces showed a peeling pattern (Fig. 3F and H).

**Fig 4:**
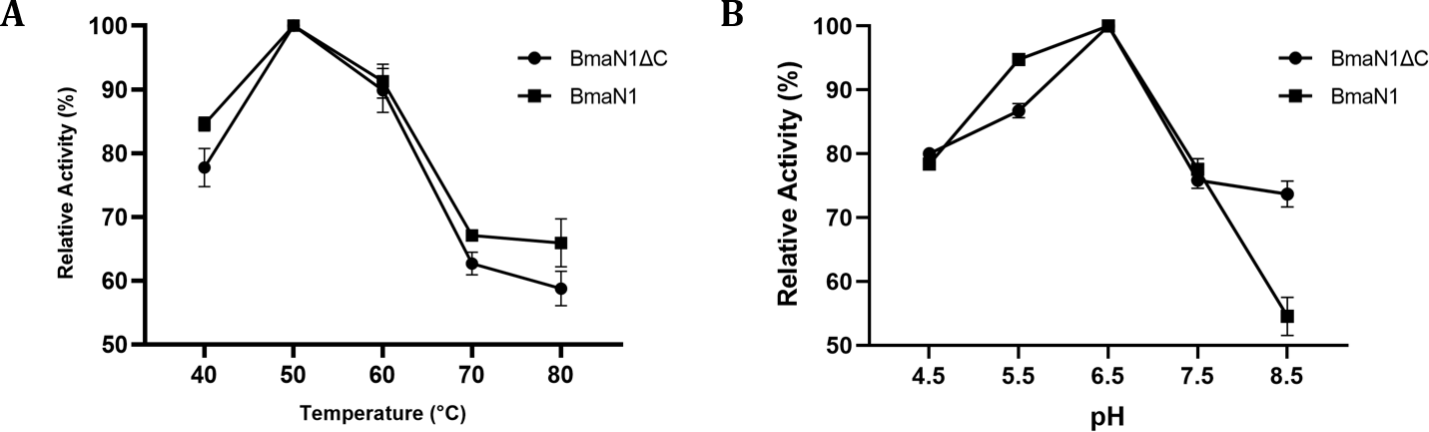
The hydrolytic activity profiles of BmaN1 and BmaN1ΔC toward soluble starch are depicted in different temperatures (**A**) and pH levels (**B**). Both proteins exhibit optimal activity at 50°C and pH 6.5.

Both BmaN1 and BmaN1ΔC showed optimal activity at 50°C and at a pH of 6.5 when using a 2% soluble starch substrate (Fig. 4A and B). They retained more than 50% of their enzymatic activity over a wide range of temperatures, ranging from 40°C to 80°C. This temperature tolerance suggests that they can remain functional under both moderate and elevated temperature conditions. Similarly, the enzymes exhibited a broad pH tolerance, retaining their activity across a wide pH spectrum from 4.5 to 8.5. This pH flexibility suggests that BmaN1 and BmaN1ΔC can function effectively in both acidic and alkaline environments.

**Fig 5:**
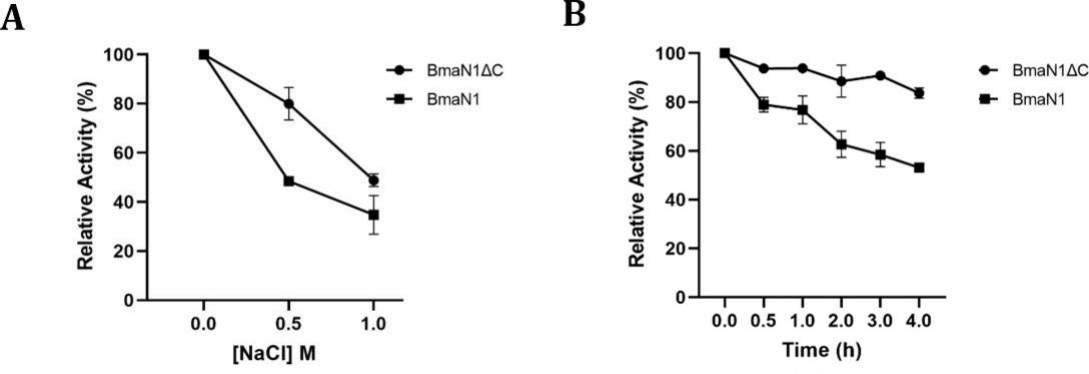
Effects of NaCl to BmaN1 and BmaN1ΔC hydrolysis activity (**A**), and BmaN1 and BmaN1ΔC stability (**B**).

**Fig 6:**
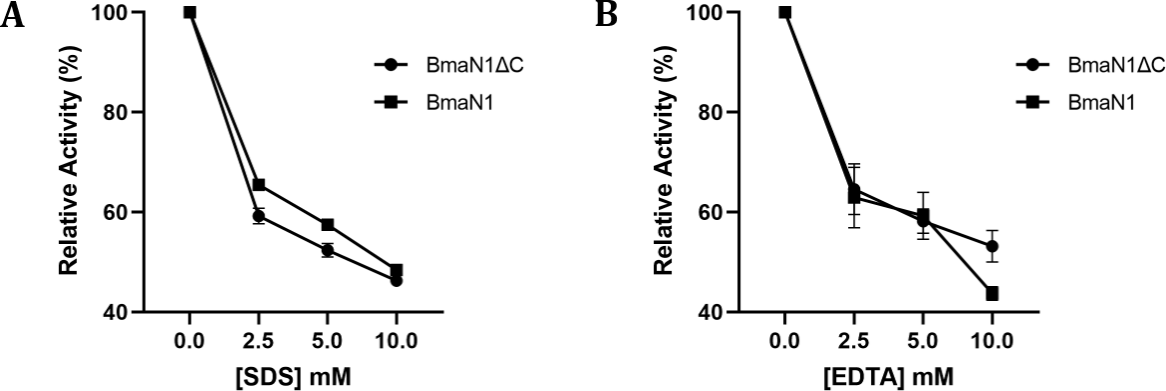
Effects of SDS (**A**) and EDTA (**B**) to BmaN1 and BmaN1ΔC hydrolysis activity.

Interestingly, despite *B. megaterium* NL3 being a marine bacterium, both enzymes demonstrated significant amylolytic activity in the absence of NaCl (Fig. 5A). BmaN1ΔC exhibited higher activity compared to BmaN1 in the presence of 0.5 and 1.0 M NaCl. However, in the presence of 2.0 and 3.0 M NaCl, both enzymes completely lost their amylolytic activity. Regarding enzyme stability, BmaN1ΔC retained 83% of its activity after 4 hours of incubation under optimal conditions, while BmaN1 displayed a comparatively lower activity, at approximately 50% (Fig. 5B). Moreover, both enzymes exhibited similar inhibition profiles following introduction to varying concentrations of SDS and EDTA (Fig. 6). The enzymes maintained over 60% activity in the presence of 2.5 mM SDS and 2.5 mM EDTA. Even at higher concentrations of SDS and EDTA, both enzymes still retained more than 40% of their activity.

The IC_50_ value of acarbose against BmaN1 and BmaN1ΔC was determined to be 34.29 and 34.7 µM, respectively. The inhibition of both enzymes by acarbose highlights their functions as an α-amylase. Furthermore, BmaN1 and BmaN1ΔC follow the Michaelis-Menten kinetics profile with soluble starch as the substrate. The kinetic parameters (Table 1) of both enzymes revealed that BmaN1 had a *k*_cat_*/K*_m_ value 1.3 higher than that of BmaN1ΔC.

**TABLE 1:** Kinetic parameters of BmaN1 and BmaN1ΔC using soluble starch

	BmaN1	BmaN1ΔC
*K*_m_ (mg.mL^−1^)	62.45 ± 3.88	101.70 ± 7.89
*k*_cat_ (s^−1^)	255.80 ± 8.80	313.17 ± 12.92
*k*_cat_/*K*_m_ (mL.mg^−1^.s^−1^)	4.09 ± 0.14	3.07 ± 0.12

## DISCUSSION

**Fig 7:**
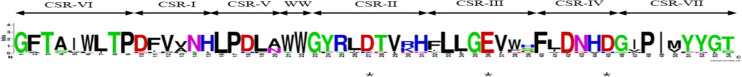
Sequence logo of CSRs of the GH13_45 subfamily. CSR-I, residues 10–15; CSR-II, residues 23–31; CSR-III, residues 32–39; CSR-IV, residues 40–45; CSR-V, residues 16–20; CSR-VI, residues 1–9; CSR-VII, residues 46–54. The two consecutive tryptophans, situated between CSR-V and CSR-II, are illustrated. The sequence of LPDlx located within CSR-V is also shown. The catalytic triad, comprising the catalytic nucleophile (No. 27, aspartic acid), the proton donor (No. 36, glutamic acid), and the transition-state stabilizer (No. 45, aspartic acid), are indicated by asterisks. The logo is constructed from the sequences of 11 characterized α-amylases belonging to the GH13_45 subfamily.

BmaN1, an α-amylase produced by *B. megaterium* NL3, is proposed for having Asp203 functions as the nucleophile, Glu231 acts as the proton donor, and His294 predicted as the transition state stabilizer (23). Moreover, BmaN1, like all characterized members of the GH13_45 subfamily, demonstrates three additional distinctive sequence-structural features: a pair of adjacent tryptophan residues, the sequence LPDlx, and a C-terminal region featuring a motif with five conserved aromatic residues (Fig. 7). The specific C-terminal sequence of BmaN1 is GFNIGYIAAVAAVYTGFGIFLYKASSKRRKEKKISQSRKKDSAS. When this C-terminal sequence was removed, it resulted in BmaN1ΔC.

Several full-length α-amylase genes have been expressed to produce extracellular proteins. Notably, α-amylase BAC from *Bacillus* sp. strain TS-23, Gt-amy from *B. thermooleovorans* NP54, and Pizzo^T^ from *G. thermoleovorans* subsp. *stromboliensis* subsp. nov have been identified as extracellular proteins (17, 33–35). Truncation of the C-terminal regions of both Gt-amy and BAC does not affect the protein folding, allowing them to function independently as α-amylases, referred to as BACΔNC and Gt-amy-T (24, 33, 34, 36). Furthermore, truncated GTA (identical to Pizzo^T^), lacking the signal peptide at the N-terminal and the transmembrane region at the C-terminal, is produced as an active intracellular protein (18). Our finding showed that the truncated form of BmaN1 (BmaN1ΔC) was produced as an active soluble protein. Taken together, removing of 45 amino acid residues from the C-terminal region of BmaN1, as well as from various α-amylases, does not affect protein folding and allows them to maintain their hydrolytic activity on soluble starch.

BmaN1 and BmaN1ΔC effectively hydrolyze soluble starch across a broad pH and temperature range, maintaining more than 50% amylolytic activity (Fig. 4A and B). Both enzymes display optimal activity under the same conditions as partially purified BaqA, specifically at a temperature of 50°C and a pH of 6.5 (21). In contrast, other α-amylase members of GH13_45, such as GTA and Pizzo^T^, show their highest activity at a temperature of 70°C and a pH of 6.0 (17, 18). Meanwhile, ASKA, ADTA, and AGXA exhibit peak activity at 60°C and a pH of 8.0 (15, 16). The members of GH13_45 meet the typical temperature and pH requirements for α-amylases used in various applications including detergents, brewing, textiles, and the paper industry (11, 37).

**Fig 8:**
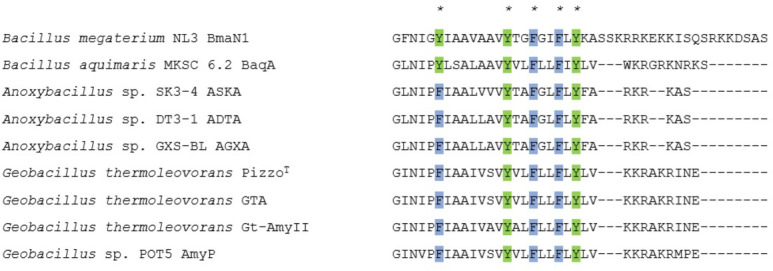
Alignment of approximately 30 residues encompassing a five-aromatic amino acid motif at the C-terminus of nine α-amylases from GH13_45. Asterisks denote all phenylalanine and tyrosine in the C-terminal region, with blue and green highlighting, respectively.

BmaN1 showed the ability to degrade both corn and cassava starches (Fig. 3B and F), but it exhibited lower efficiency compared to Gt-amy and other enzymes classified as raw starch-digesting α-amylases (RSDA) (24). Interestingly, BmaN1, along with α-amylases of the GH13_45 subfamily, lacks the starch-binding domain (SBD) despite their ability to degrade raw starch granules. In addition to the SBD, amylolytic enzymes can also bind to the substrate's surface through what are known as surface binding sites (SBSs). These SBSs are distinct from the enzyme's active site and play a critical role in guiding and targeting the substrate toward the active site groove (38). SBSs are typically formed by aromatic amino acids located on the enzyme's surface (38–41). Both BmaN1 and α-amylases within the GH13_45 subfamily possess a shared motif, comprising a conserved C-terminal region of about 30 residues. This segment contains five aromatic residues, including phenylalanine and tyrosine, which are thought to be crucial for binding and degrading raw starch granules (41). The C-terminal amino acids sequence of BmaN1 features a motif comprising five conserved aromatic residues, consistent with the GH13_45 family (Fig. 8). Based on the observed activity of BmaN1∆C compared to BmaN1 in degrading corn and cassava granules (Fig. 2 and 3), it can be inferred that the extended C-terminal region of BmaN1 plays a role in sequestering and rendering starch more accessible for subsequent processing and hydrolysis. Similar phenomena were observed with the extracellular native α-amylase Pizzo^T^, which exhibited high digestibility the raw corn starch granules at concentrations ranging from 15% to 20%. Specifically, Pizzo^T^ was found to adsorb onto raw corn starch at a rate of 97% after 10 minutes of incubation (17). These observations are consistent with our results indicating that the C-terminal region of recombinant BmaN1 is involved in binding to and degrading raw starch. Nevertheless, a site directed mutagenesis study is required to elucidate the role of the five aromatic residues in this C-terminal region.

**Fig 9:**
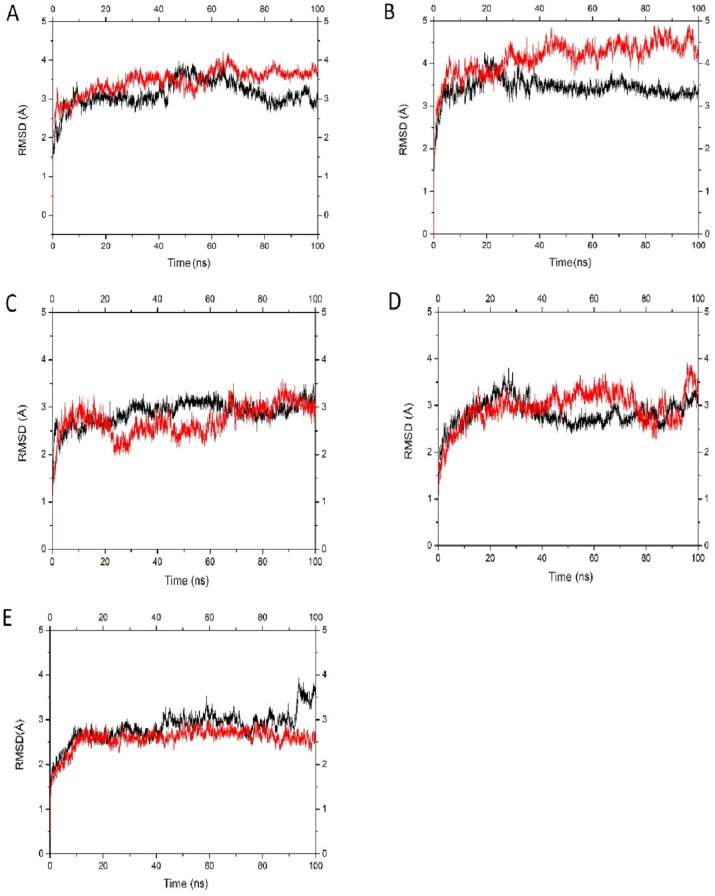
Profile of root mean square deviations (RMSD) of BmaN1 (red) and BmaN1ΔC (black) structures at various salt concentrations: 0.0 M (**A**), 0.5 M (**B**), 1.0 M (**C**), 2.0 M (**D**), and 3.0 M (**E**).

**Fig 10:**
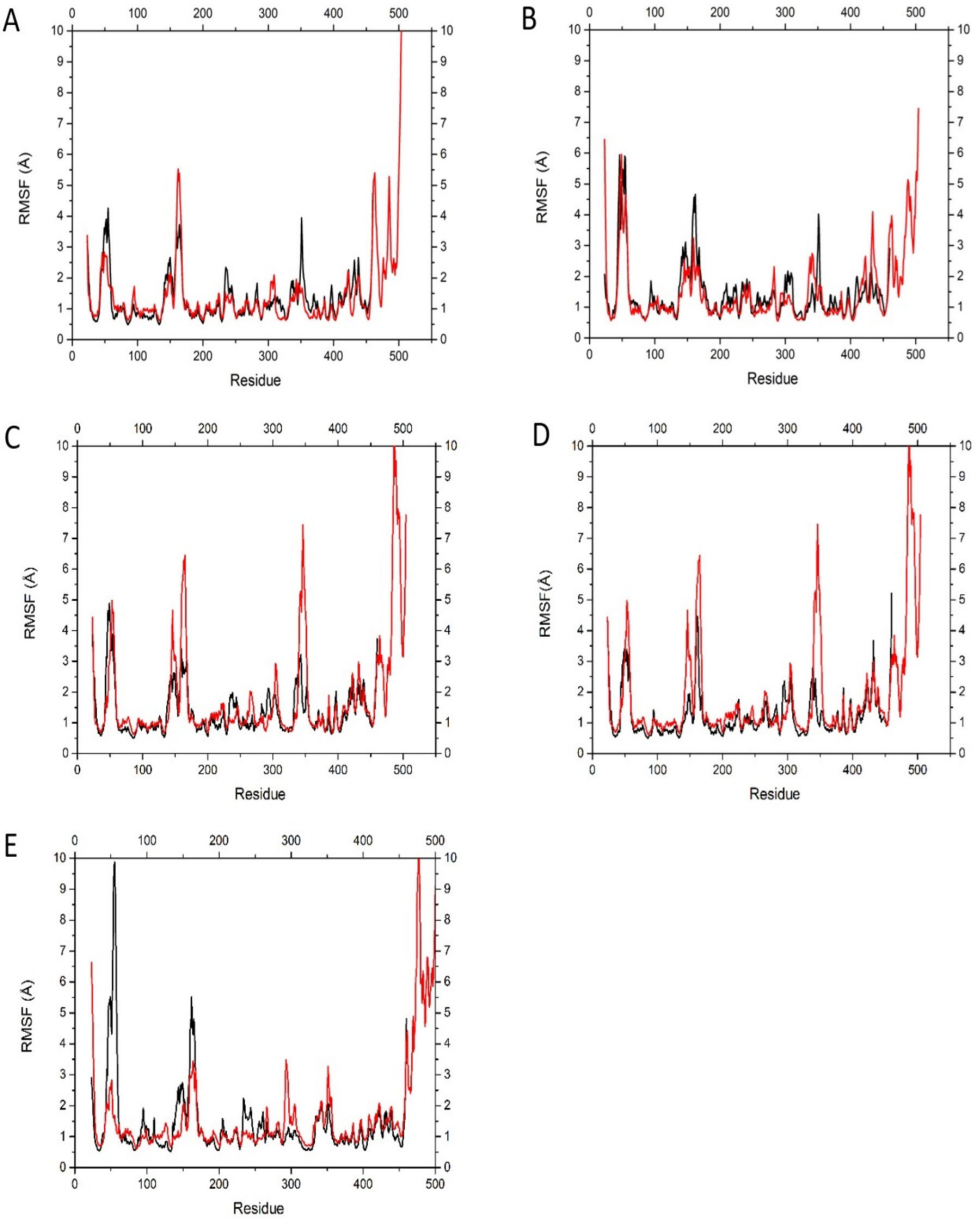
Profiles of root mean square fluctuation (RMSF) of BmaN1 (red) and BmaN1ΔC (black) structures at various salt concentrations: 0.0 M (**A**), 0.5 M (**B**), 1.0 M (**C**), 2.0 M (**D**), and 3.0 M (**E**).

**Fig 11:**
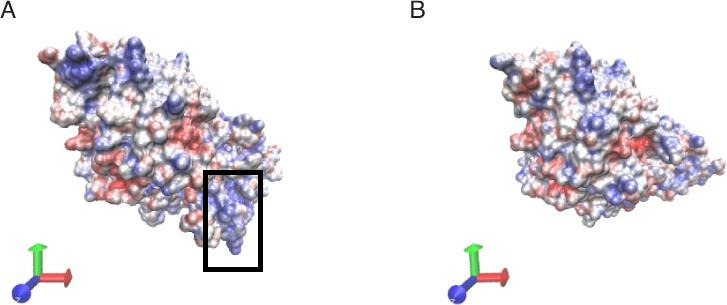
Electrostatic surface representations of BmaN1 (**A**) and BmaN1ΔC (**B**) (note: the C-terminal of BmaN1 showed in box).

Both BmaN1 and BmaN1ΔC displayed resilience toward SDS, EDTA, and NaCl (Fig. 6 and Fig. 5A). According to molecular dynamics (MD) simulations, both BmaN1 and BmaN1∆C demonstrate robust stability at the optimal temperature in the presence of 0.5–3.0 M NaCl (Fig. 9). This is evident from the root mean square deviation (RMSD) values for both enzymes, which are consistently remaining below 5 Å. These values are comparable to those observed for Cel5R (a halotolerant cellulase from a soil metagenome) and TfCel5A (a non-halotolerant cellulase from *Thermobifida fusca*) during the stabilization of their integrated structures in various NaCl solutions based on MD simulations (42). To better understand how ligand binding affects protein flexibility, we calculated the flexibility of each residue using root mean square fluctuation (RMSF) (43). Notably, the C-terminal region (residues 460–504) of BmaN1 exhibits the highest fluctuations in the presence of 0.5–3.0 M NaCl (Fig. 10). Elevated RMSF values in the C-terminal region of BmaN1 suggest that the protein remains highly flexible even when unliganded, potentially impacting the stability of the BmaN1 structure. Furthermore, the predicted helical C-terminal region of BmaN1 exhibits a positive surface potential (Fig. 11), likely due to repulsive forces arising from the similar positive charges among BmaN1 residues. Reducing these positive charges at the C-terminal of BmaN1∆C contributes to its higher stability in salt conditions. In line with the findings from molecular dynamics simulations, the deletion of the C-terminal region in BmaN1ΔC not only reduces positive charges but also decreases flexibility, thereby enhancing the enzymatic activity of BmaN1∆C in harsh environments when compared to its full-length counterpart.

### Conclusion

BmaN1 exhibits versatility by effectively hydrolyzing soluble starch across a wide range of pH values, temperatures, SDS, EDTA, and salt concentrations. Additionally, it has an ability to hydrolyze both corn and cassava granules at temperatures lower than those commonly used in industrial processes. More importantly, this study has shed light that the presence of amino acids at C-terminal region of BmaN1 significantly enhances its binding and degrading the raw starch.
